# The spinal microglial IL-10/β-endorphin pathway accounts for cinobufagin-induced mechanical antiallodynia in bone cancer pain following activation of α7-nicotinic acetylcholine receptors

**DOI:** 10.1186/s12974-019-1616-z

**Published:** 2020-02-29

**Authors:** Evhy Apryani, Usman Ali, Zi-Ying Wang, Hai-Yun Wu, Xiao-Fang Mao, Khalil Ali Ahmad, Xin-Yan Li, Yong-Xiang Wang

**Affiliations:** Shanghai Jiao Tong University School of Pharmacy, 800 Dongchuan Road, Shanghai, 200240 China

**Keywords:** Cinobufagin, Microglia, IL-10/β-endorphin pathway, α7-nicotinic acetylcholine receptor (α7-nAChR)

## Abstract

**Background:**

Cinobufagin is the major bufadienolide of *Bufonis venenum* (Chansu), which has been traditionally used for the treatment of chronic pain especially cancer pain. The current study aimed to evaluate its antinociceptive effects in bone cancer pain and explore the underlying mechanisms.

**Methods:**

Rat bone cancer model was used in this study. The withdrawal threshold evoked by stimulation of the hindpaw was determined using a 2290 CE electrical von Frey hair. The β-endorphin and IL-10 levels were measured in the spinal cord and cultured primary microglia, astrocytes, and neurons.

**Results:**

Cinobufagin, given intrathecally, dose-dependently attenuated mechanical allodynia in bone cancer pain rats, with the projected *E*_max_ of 90% MPE and ED_50_ of 6.4 μg. Intrathecal cinobufagin also stimulated the gene and protein expression of IL-10 and β-endorphin (but not dynorphin A) in the spinal cords of bone cancer pain rats. In addition, treatment with cinobufagin in cultured primary spinal microglia but not astrocytes or neurons stimulated the mRNA and protein expression of IL-10 and β-endorphin, which was prevented by the pretreatment with the IL-10 antibody but not β-endorphin antiserum. Furthermore, spinal cinobufagin-induced mechanical antiallodynia was inhibited by the pretreatment with intrathecal injection of the microglial inhibitor minocycline, IL-10 antibody, β-endorphin antiserum and specific μ-opioid receptor antagonist CTAP. Lastly, cinobufagin- and the specific α-7 nicotinic acetylcholine receptor (α7-nAChR) agonist PHA-543613-induced microglial gene expression of IL-10/β-endorphin and mechanical antiallodynia in bone cancer pain were blocked by the pretreatment with the specific α7-nAChR antagonist methyllycaconitine.

**Conclusions:**

Our results illustrate that cinobufagin produces mechanical antiallodynia in bone cancer pain through spinal microglial expression of IL-10 and subsequent β-endorphin following activation of α7-nAChRs. Our results also highlight the broad significance of the recently uncovered spinal microglial IL-10/β-endorphin pathway in antinociception.

## Introduction

*Bufonis venenum* (Chansu) is a traditional Chinese medicine that is prepared from the dried white secretion of the auricular glands and the skin glands of *Bufo gargarizans Cantor* or *Bufo melanostictus Schneider*. Being listed in the Chinese Pharmacopeia, *Bufonis venenum* has been used as a therapeutic agent in Asian countries for centuries to treat cardiovascular diseases, inflammatory diseases, cancers and chronic pain [12, 70, 80, 87, 88, 91]. The major active components in *Bufonis venenum* are bufadienolides, such as cinobufagin, bufalin, resibufogenin, cinobufotalin, bufotenine, bufotenidine, and bufobutanoicacid, the first three of which account for approximately 10% of the dry weight of toad venom [43, 46, 47, 84, 86]. The antiinflammatory and antinociceptive effects of *Bufonis venenum* and its purified bufadienolides have been extensively studied over the past years. Gavage and intraperitoneal administration of *Bufonis venenum*, cinobufagin and bufalin produced marked antinociception in the hot-plate, formalin and acetic acid writing tests [74, 81, 82]. Interestingly, *Bufonis venenum* has remarkable analgesic effect in patients with cancer pain including that from bone metastasis [22, 50], while it has been also served as an anti-tumor agent [2, 12, 25, 48]. Preclinical studies showed that multiple daily intraperitoneal injections of *Bufonis venenum* (referred as to cinobufagin in the original paper) exerted mechanical antiallodynia and thermal antihyperalgesia in a mouse model of paw cancer pain [9, 10].

However, the mechanisms underlying *Bufonis venenum*- and its effective ingredients-induced analgesia especially in cancer pain remain unclear. It was postulated that *Bufonis venenum* produced antinociception through inhibition of neuronal adenosine triphosphatase sodium-potassium pump or Na^+^-K^+^ ATPase [72], as its effective ingredients bufadienolides are a group of steroid hormones, which, like ouabain, inhibit Na^+^-K^+^ ATPase [11, 17, 18, 41] leading to antinociception [15, 68]. Also, it was recently proposed that cinobufagin exerted antinociception via activation of the α-7 nicotinic acetylcholine receptor (α7-nAChR), since cinobufagin antinociception in the hot-plate, formalin and acetic acid writing tests were reversed by intraperitoneal administration of the specific α7-nAChR antagonist methyllycaconitine and intrathecal injection of the α7-nAChR gene silencer siRNA/α7-nAChR [82]. The α7-nAChR, which is expressed in glial cells and closely associated with inhibition of neuroinflammation, is a potential target molecule for the treatment of chronic pain [1, 14, 27, 38, 62].

It is also controversial whether the inhibition of the proinflammatory cytokine expression or stimulation of the opioid peptide expression is responsible for *Bufonis venenum* or its effective ingredients-induced antinociception. Treatment with cinobufagin in human monocyte-derived dendritic cells potently inhibited LPS-induced maturation and production of proinflammatory cytokines including interleukin (IL)-1β, IL-6, and tumor necrosis factor (TNF)-α [80]. Intraperitoneal injection of bufalin also reduced carrageenan-induced edema, and inhibited NF-κB activation and downregulated the expression of inducible nitric oxide synthase, cyclooxygenase-2, IL-1β, IL-6, and TNF-α in the carrageenan-injected paw tissues [74]. In addition, cinobufagin injection in the dorsal root ganglion inhibited NF-κB activation and proinflammatory cytokine expression [82]. These findings suggested that *Bufonis venenum* induced antinociception via inhibition of NF-κB activation and subsequent expression of inflammatory cytokines [74, 80, 82]. However, administration of *Bufonis venenum* promoted the β-endorphin and corticotropin-releasing factor (CRF) levels in plasma, and immunostaining of β-endorphin and μ-opioid receptors in the tumor xenograft tissues of paw cancer pain mice; treatment with *Bufonis venenum* also stimulated the expression of β-endorphin and CRF in cultured primary lymphocytes [9, 10]. Moreover, the antinociceptive effect of *Bufonis venenum* or bufalin was blocked by the pretreatment with the opioid receptor antagonist naloxone or its peripheral form naloxone methiodide [9, 10, 74]. These studies suggested that *Bufonis venenum* and its effective ingredients produced antinociception through the β-endorphin/opioid receptor pathway [9, 10].

Cinobufagin is probably the most abundant bufadienolide in *Bufonis venenum* and has displayed significant analgesic effect [35, 43, 46, 47, 84, 86]. Therefore, it was selected in this study to evaluate the antinociceptive effects of *Bufonis venenum* in cancer pain and explore the underlying mechanisms. We recently discovered that microglia-derived IL-10 in neuropathic pain produced mechanical antiallodynia and thermal antihyperalgesia via autocrine secretion of β-endorphin which interacted with neuronal μ-opioid receptors [77, 78]. Futhermore, we also uncovered that the glucagon-like peptide-1 (GLP-1) receptor agonist exenatide and GPR40 agonist GW9508 produced mechanical antiallodynia and thermal antihyperalgesia in neuropathic rats through autocrine glial secretion of IL-10 and subsequent β-endorphin [52, 79]. Thus, we postulated that cinobufagin may produce antinociception by triggering the microglial IL-10/β-endorphin pathway after activation of α7-nAChRs. We first established the rat model of bone cancer pain and assessed the mechanical antiallodynic effect of cinobufagin given intrathecally. We then observed the stimulatory effects of cinobufagin on the expression of IL-10 and subsequent β-endorphin in the spinal cords of bone cancer rats and cultured spinal primary microglial cells. Furthermore, we attempted to illustrate whether spinal microglial expression of IL-10/β-endorphin was causally associated with cinobufagin-induced mechanical antiallodynia. Lastly, we tested whether cinobufagin-induced microglial IL-10/β-endorphin expression and mechanical antiallodynia were α7-nAChR-dependent.

## Materials and methods

### Drugs and reagents

Cinobufagin was purchased from ChemFaces (Wuhan, Hubei, China) with ≥ 98% purity determined by ^1^H-NMR, and SP600125, SB203580 and U0126 were obtained from Selleck Chemicals (Houston, TX, USA). PHA-543613, ouabain octahydrate, and methyllycaconitine citrate were obtained from Sigma-Aldrich (St. Louis, MO), Acros Organics (NJ, USA) and ApexBio (Shanghai, China), while minocycline and CTAP were purchased from Yuanye Biotech (Shanghai, China) and Tocris (Bristol, UK), respectively. α-Helical CRF (9-41) was synthesized with the peptide contents of 98% in Shanghai TASH Biotechnology Co. (Shanghai, China). The rabbit β-endorphin antiserum and recombinant rat IL-10 antibody were purchased from Abcam (Cambridge, UK) and PeproTech (NJ, USA), respectively. Cinobufagin was dissolved in 10% dimethyl sulfoxide (DMSO) and 20% polyethylene glycol (PEG400) in 0.9% normal saline for intrathecal injection and in 0.1% DMSO for cell culture. α-Helical CRF (9-41) was dissolved in 30% DMSO and 70% PEG400. All other drugs or reagents were dissolved in normal saline.

### Animals

Male or female neonatal (1 day old) and adult (8–10 weeks old) Wistar rats were purchased from the Shanghai Experimental Animal Institute for Biological Sciences (Shanghai, China). The adult rats were housed in the plastic cages in the animal room with temperature- and humidity-controlled environment on a 12-h-light/dark cycle and acclimatized to the laboratory environment for 2–3 days prior to experiments, with food and water ad libitum. The animal procedure protocols were approved by the Animal Care and Welfare Committee of the Shanghai Jiao Tong University (Shanghai, China) and followed the regulatory animal care guidelines of the US National Institutes of Health.

### Rat model of bone cancer pain

The rat bone cancer pain model was induced as described previously [24, 31]. Walker 256 carcinoma cells (4 × 10^5^) in 10 μL of the phosphate buffer solution were implanted directly into the tibia cavity of female or male anesthetized rats using a 10-μL microinjection syringe. The injection site was sealed using the aseptic bone wax to prevent the spreading of the carcinoma cells to the surrounding soft tissue. The wound was covered with the penicillin powder and closed. After recovery from the inoculation surgery and anesthesia, the rats were returned to their home cages.

### Intrathecal injection

The direct intrathecal injection was performed by holding the rat securely in one hand by the pelvic girdle and inserting a 25-gauge × 1 in. needle, connected to a 25-μL Hamilton syringe, into the tissues between the dorsal aspects of L5 and L6, perpendicular to the vertebral column. The tip of the needle was kept at the injection site for approximately 15 s to ensure the delivery of the solution. The quality of each injection was ensured by the observation of an injection-induced tail-flick [53]. The method was validated by using the lidocaine paralysis method [52] and extensively practiced in house.

### Behavioral assessment of mechanical allodynia

The assessment of withdrawal thresholds in the contralateral and ipsilateral hindlimbs of bone cancer pain rats was conducted approximately 3 weeks after the inoculation of Walker 256 carcinoma cells surgery as described by Wu et al. [77, 78]. Bone cancer pain rats were randomly assigned to the experimental groups (*n* = 6 in each group) and the behavior observations were performed in a blind manner to the experimental conditions. Rats were placed in the metal grid covered with a plastic box and allowed to be habituated to the testing environment for at least 30 min before conducting the behavioral assessment. The withdrawal threshold evoked by stimulation of the hindpaw was determined using a 2290 CE electrical von Frey hair (IITC Life Science, Woodland Hills, CA, USA) while the rat stood on a metal grid. The monofilament, which generated a force that ranged from 0.1 to 90 g, was applied in the foot pad with increasing force until the rat suddenly withdraws its hindlimb. The lowest force evoking a withdrawal response was considered the threshold, which was averaged from triplicate measurement in an interval of approximately 1 min.

### Primary cell cultures

The spinal cord, extracted from the 1-day-old neonatal rats, was minced and digested in 0.05% trypsin for 10 min. The dispersive cells after centrifugation were thereafter suspended in Dulbecco’s modified Eagle’s medium (DMEM) supplemented with 10% (v/v) fetal bovine serum (FBS), penicillin (100 U/mL), and streptomycin (100 μg/mL). The cells were then plated to poly-l-lysine (100 μg/mL)-coated flask and incubated at 37 °C with 5% CO_2_ in the incubator. To obtain neuronal cells, the medium after 1.5 h of incubation was changed to neurobasal containing B27 supplement and 0.5 mM glutamine for further culture. The purity of neuronal cells, determined by the neuronal nuclear antigen (NeuN) immunoreactivity [21], was more than 85%. All experiments were initiated 5–6 days after plating.

For glial cell cultures, cells were seeded into 75-cm^2^ tissue culture flasks pre-coated with poly-l-lysine (1 × 10^7^ cells/flask), and cultured at 37 °C. After 8 days of culture, microglial cells were obtained as a floating cell suspension after shaking the flask at 260 rpm for 2 h. Aliquots were transferred to plates and unattached cells were removed by washing with serum-free DMEM. Harvested microglial cells exhibited a purity > 95%, as determined by the CD11b (OX42) immunoreactivity [77, 78]. All experiments were initiated 12–24 h after plating. To obtain astrocytes, the flask after 11 days of culture was shaken for 2 h and then the aliquots were removed. The cells were washed using PBS and incubated with 10 mL of 0.05% trypsin-ethylenediamine tetraacetic acid (Invitrogen, Grand Island, NY, USA) for 15 min to separate the oligodendrocytes. The trypsin was neutralized using 10 mL complete DMEM and the floating cell suspensions were discarded. A nearly intact layer of astrocytes in the bottom of the flasks were then trypsinized and subcultured conventionally. Prepared astrocytes showed a purity 90% as determined by the GFAP immunoreactivity [77, 78]. All experiments were initiated 12–24 h after plating.

### RNA isolation and quantitative real-time PCR (qRT-PCR)

The spinal lumbar enlargements (L3-L5) were isolated from the bone cancer rats 1 h after the testing drug administration. The spinal homogenates and primary culture cells were mechanically homogenized in Trizol (Invitrogen) and the obtained total RNAs were reversely transcribed into cDNA using the ReverTraAce qPCR RT-kit (Toyobo, Osaka, Japan) according to the manufacturer’s instruction. qRT-PCR amplification was performed in the Mastercycler ep realplex (Eppendorf, Hamburg, Germany) using the RealmasterMix (SYBR Green I) (Toyobo) in combination with the following primer sets: 5’CCAAGGTCATCCATGACGAC-3′ and 5′-TCCACAGTTCTGAGTGGC-3′ for GAPDH, 5′-CCTATCGGGTGGAGCACTTC-3′ and 5′-TGGCTCTTCTCGGAGGTCAT-3′ for the β-endorphin precursor proopiomelanocortin (POMC) [21], 5′-GGCTCAGCACTGCTATGTTGCC-3′ and 5′-AGCATGTGGGTCTGGCTGACTG-3′ for IL-10 (NM_012854.2), 5′-ACTGCCTGTCCTTGTGTTCC-3′ and 5′-CCAAAGCAACCTCATTCTCC-3′ for the dynorphin precursor prodynorphin (PDYN) [45]. For the relative quantification, the target gene expression was determined by the (2^−∆∆Ct^) method after normalization to the gene of GAPDH CT values.

### β-Endorphin and IL-10 measurements

The spinal cord, extracted from the bone cancer rats 1 h after the testing drug injection, was homogenized at 4000 rpm for 15 s with a homogenizer (Fluko Equipment) in 10 mM Tris-HCl (1 g of tissue/5 mL) and centrifuged at 4000 rpm in 4 °C for 15 min. For primary cells, cell culture supernatants were collected after treating with testing drugs for 2 h and then centrifuged at 5000 rpm at 4 °C for 5 min. The supernatants after centrifuge were prepared as the protein samples for ELISA. The protein concentrations in the spinal homogenates or cultured cell aliquots were measured by using the standard bicinchoninic acid protein assay (Beyotime Institute of Biotechnology, Jiangsu, China). The levels of IL-10 (eBioscience, Waltham, MA, USA) and β-endorphin (Phoenix Pharmaceuticals, CA, USA) were measured by using the enzyme-lined fluorescent immunoassay kits according to the manufacturer’s instruction. A fluorescence microplate reader (Thermo Labsystem) was used to measure the relative fluorescence values and the concentration of IL-10 or β-endorphin was calculated by a calibration curve performed at the same time. The assays were validated with the linear range of 1–10,000 pg/mL for IL-10 and 1–1000 pg/mL for β-endorphin. The β-endorphin ELISA kit had cross-reactivity with α-endorphin (100%) and γ-endorphin (60%), but not with met-enkephalin (0%) or leu-enkephalin (0%), based on the manufacturer’s information.

### Data calculation and statistical analysis

The withdrawal threshold data was converted to the percentage of the maximum possible effect (% MPE) by using the formula: (post-drug threshold in ipsilateral hindpaw − pre-drug threshold in ipsilateral hindpaw)/(post-drug threshold in contralateral hindpaw − pre-drug threshold in ipsilateral hindpaw) × 100%. The % MPE values approximated to 100 indicate normal mechanical thresholds (i.e., near contralateral thresholds), while values approximated to 0 indicate mechanical allodynia. For the analysis of the dose-response curve, the following parameters were calculated by fitting non-linear least-squares curves to the relation *Y* = *a* + *bx*, where *x* = [*D*]^*n*^/(ED_50_^*n*^ + [*D*]^*n*^), i.e., the minimum effect, maximum effect (*E*_max_), half-effective concentration (ED_50_) and Hill coefficient (*n*). The values of ED_50_ and *b* (*E*_max_) were projected by yielding a minimum residual sum of squares of deviations from the theoretical curve [73].

The results are expressed as the mean ± standard error of mean (SEM). Unpaired and two-tailed Student *t* test, and one-way and repeated-measures two-way ANOVA were used to generate the statistical significance values. The post hoc Student–Newman–Keuls test was performed when the effect of the drug [dose] (for one-way ANOVA, the factor was drug [dose]; for two-way ANOVA, the factors were drug [dose], time and their interaction) was statistically significant. Probability values were two-tailed and the statistical significance criterion *p* value was 0.05. The statistical analysis and dose-response projection were performed by using Prism (version 7.01; GraphPad Software, Inc., San Diego, CA, USA).

## Results

### Cinobufagin produced mechanical antiallodynia and stimulated spinal expression of IL-10 and β-endorphin

**Fig. 1:**
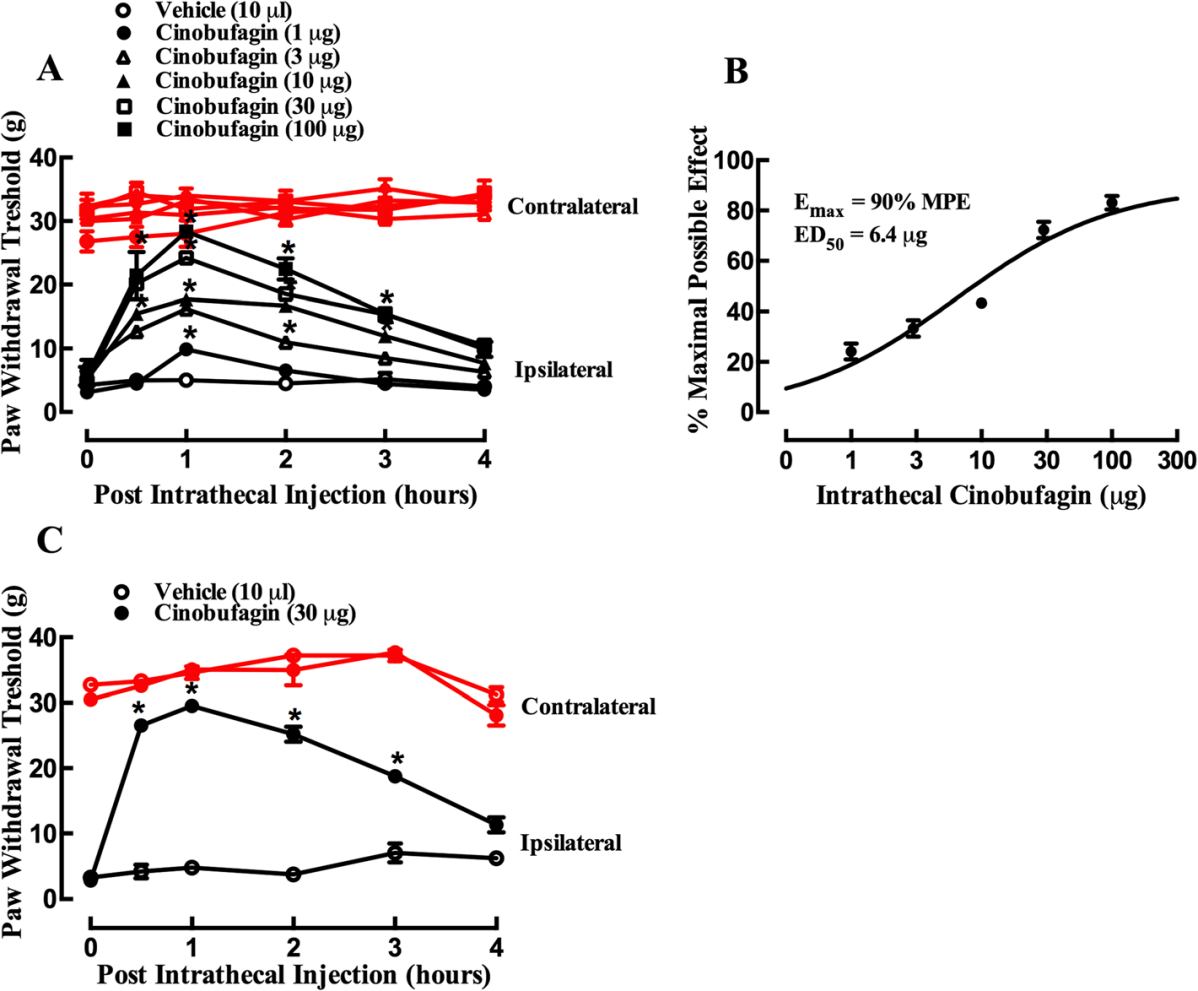
Inhibitory effects of cinobufagin, given intrathecally, on mechanical allodynia in the rat model of bone cancer pain. The rats were inoculated with cancer cells for approximately 3 weeks and their mechanical thresholds were then measured by using electric von Frey filaments in both the contralateral and ipsilateral hindpaws. **a** Female bone cancer pain rats received single intrathecal administration of saline or cinobufagin (1, 3, 10, 30, or 100 μg). **b** Dose-response analysis of cinobufagin on mechanical allodynia in the ipsilateral hindpaws of female bone cancer pain rats 1 h after its injection, best projected by the non-linear least-squares method. **c** Male bone cancer pain rats received single intrathecal administration of saline or cinobufagin (30 μg). The data are presented as means ± SEM (*n* = 6 per group). The asterisk denotes statistical significance (*p* < 0.0001) compared to the saline control group, by repeated-measured two-way ANOVA followed by the post hoc Student–Newman–Keuls test

Mechanical allodynia started approximate 2 weeks after cancer cell tibia inoculation in rats and reached peak around 3 weeks after inoculation and remained consistent thereafter up to 4 weeks in our observation. The mechanical antiallodynic effects of cinobufagin were first assessed in the rat model of bone cancer pain by using von Frey filaments. A total of 36 female bone cancer pain rats were divided into six groups and received intrathecal injection of the vehicle (10% DMSO and 20% PEG400 in saline, 10 μL) and cinobufagin (1, 3, 10, 30, and 100 μg), respectively. The paw withdrawal responses to mechanical stimulation were observed before and 0.5, 1, 2, and 4 h after drug administration in both the contralateral and ipsilateral hindpaws. As shown in Fig. 1a, intrathecal injection of cinobufagin up to 100 μg did not significantly alter withdrawal thresholds in the contralateral hindpaws during 4 h of observation. However, it alleviated mechanical allodynia in the ipsilateral hindpaws in a time-dependent manner, with the peak effect occurring between 30 and 60 min and the action duration longer than 3 h (*p* < 0.0001, by repeated-measured two-way ANOVA followed by the post hoc Student–Newman–Keuls test). Cinobufagin-induced mechanical antiallodynia was also dose-dependent, with the *E*_max_ value of 90% MPE and ED_50_ value of 6.4 μg (95% confidence limits of 2.6 to 15.4 μg) as projected from the withdrawal thresholds in the ipsilateral hindpaws 1 h after injection (Fig. 1b). There was no obvious sedation or impaired locomotor activity observed during the study. However, intrathecal injection of more than 250 μg of cinobufagin caused abnormal behaviors including restless movements and tonic convulsion. In addition, the mechanical antiallodynic effects of cinobufagin were also evaluated in male bone cancer rats. As shown in Fig. 1c, intrathecal injection of cinobufagin (30 μg) significantly alleviated mechanical allodynia in the ipsilateral hindpaws of male rats (*p* < 0.0001, by repeated-measured two-way ANOVA followed by the post hoc Student–Newman–Keuls test) by a similar level to that of female rats, although it did not significantly alter withdrawal thresholds in the contralateral hindpaws.

**Fig. 2:**
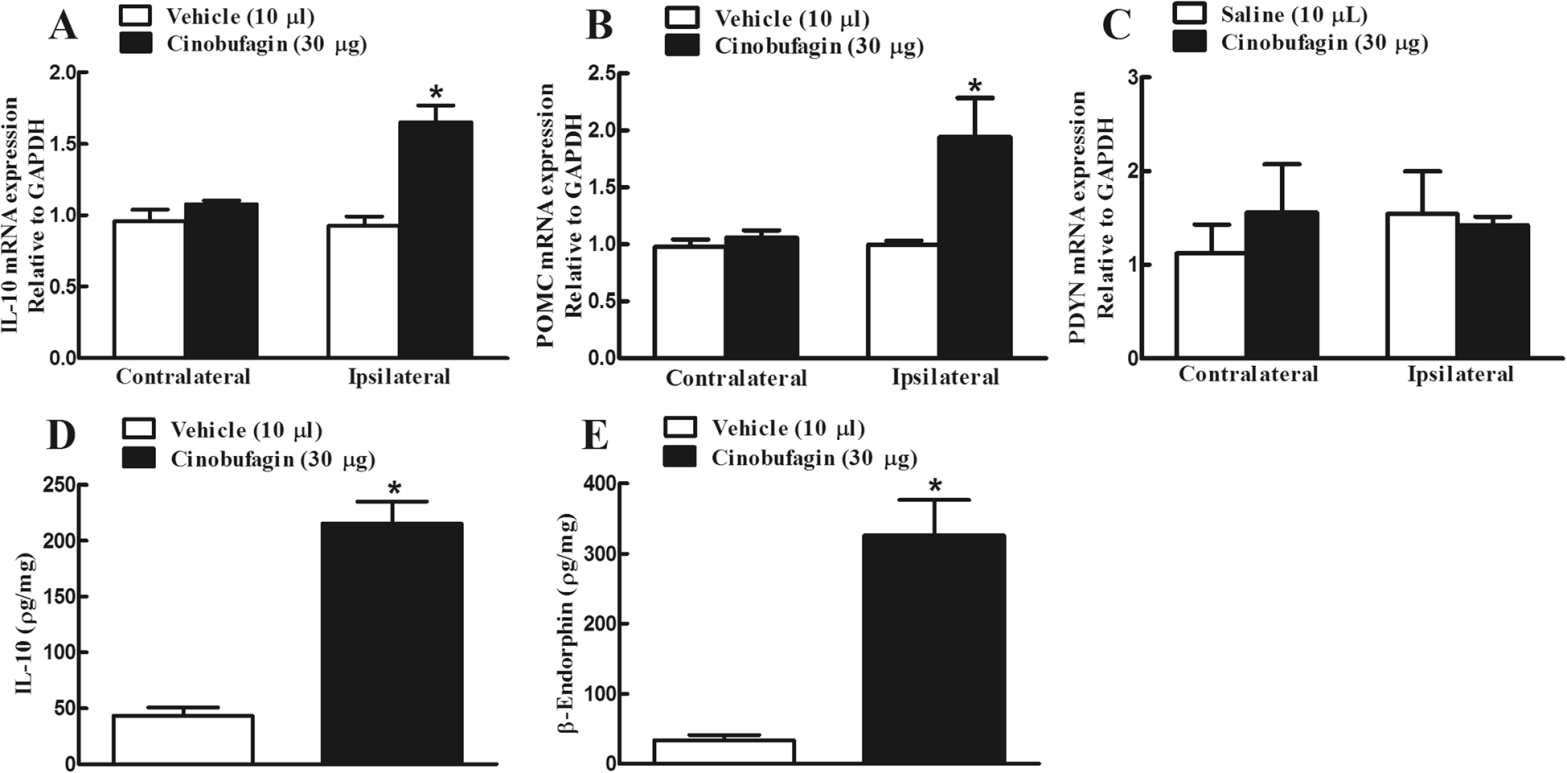
Effects of cinobufagin on spinal gene (**a**–**c**) and protein (**d**, **e**) expression of IL-10, β-endorphin and dynorphin A in bone cancer pain rats. The spinal lumbar enlargement (L3-L5) of female bone cancer pain rats were collected 1 h after intrathecal injection of cinobufagin. The mRNA expression of IL-10, the β-endorphin precursor proopiomelanocortin (POMC) and dynorphin precursor prodynorphin (PDYN), and protein expression of IL-10 and β-endorphin were measured by using qRT-PCR and commercial fluorescent immunoassay kits, respectively. The data are presented as means ± SEM (*n* = 6 per group). The asterisk denotes statistical significance (*p* < 0.05) compared to the saline control group, by unpaired and two-tailed Student *t* test

Two groups of female bone cancer rats received intrathecal injection of the vehicle (10 μL) and cinobufagin (30 μg). The spinal lumbar enlargements were removed 1 h after intrathecal administration of cinobufagin. Intrathecal cinobufagin injection significantly upregulated the mRNA expression of IL-10 (Fig. 2a) and POMC (Fig. 2b), measured by using qRT-PCR, in the ipsilateral spinal cords (*p* < 0.01, by unpaired and two-tailed Student *t* test). In contrast, intrathecal cinobufagin did not significantly affect the PDYN mRNA expression (Fig. 2c).

Furthermore, the baseline IL-10 and β-endorphin levels were 43.1 ± 7.8 and 33.3 ± 7.9 pg/mL in the spinal homogenates from the vehicle control rats, measured by using the commercial fluorescent immunoassay kits. Intrathecal cinobufagin (30 μg) injection significantly increased the protein expression of IL-10 by 5.0-fold and β-endorphin by 10.1-fold, respectively (*p* < 0.01 by unpaired and two-tailed Student *t* test; Fig. 2d, e).

### Cinobufagin specifically stimulated microglial expression of IL-10 and subsequent β-endorphin

**Fig. 3:**
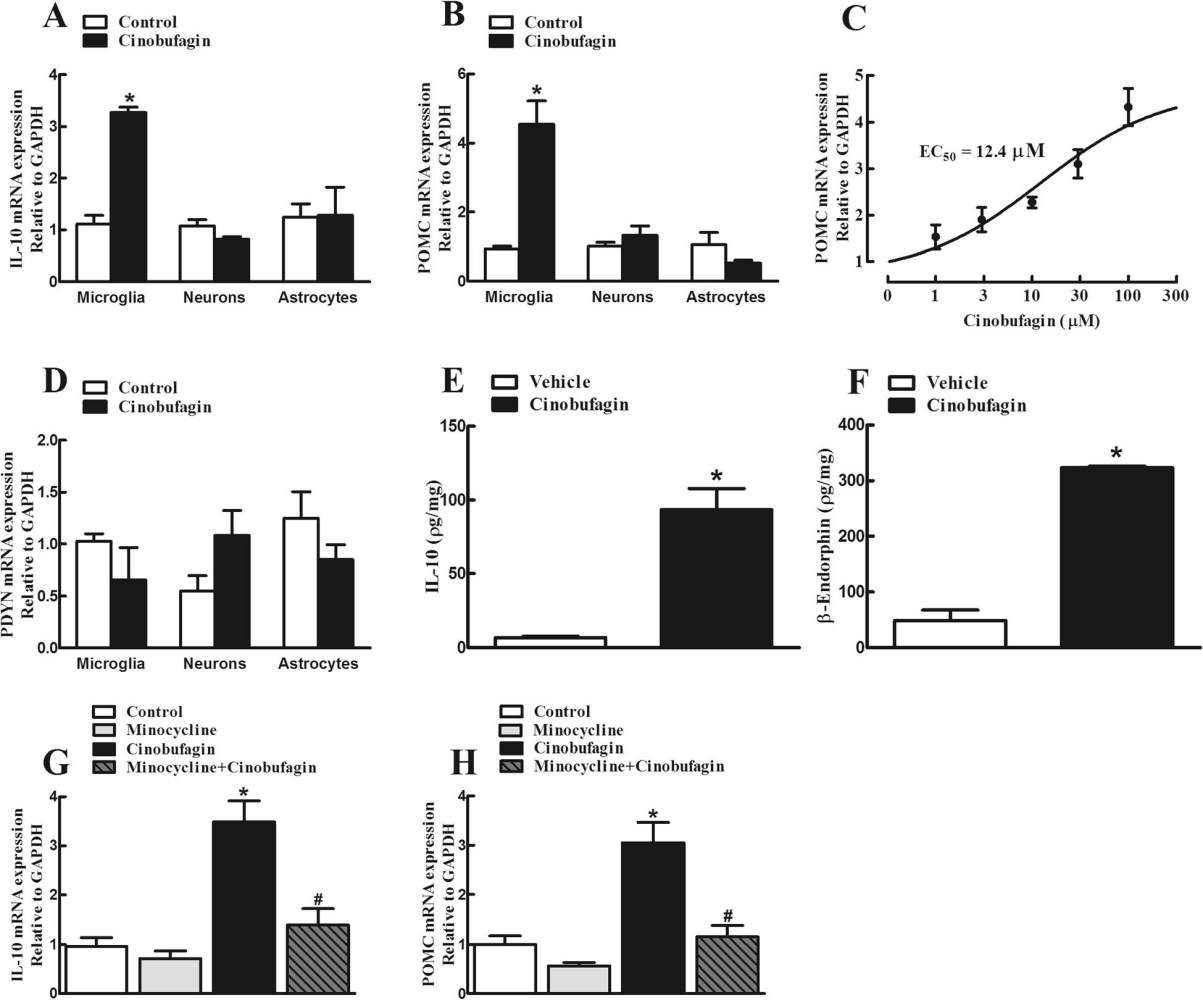
Effects of cinobufagin on the gene (**a**–**d**) and protein (**e**, **f**) expression of IL-10, the β-endorphin precursor proopiomelanocortin (POMC) and dynorphin precursor prodynorphin (PDYN) in the primary cultures of spinal microglia, neurons, and astrocytes. **g**, **h** The blockade effect of the microglial inhibitor minocycline on cinobufagin-induced gene overexpression of IL-10 and POMC. The primary culture cells, originated from the spinal cords of 1-day-old neonatal rats, were collected 2 h after cinobufagin incubation. For the blockade study, minocycline was incubated 1 h prior to cinobufagin treatment. The mRNA expression of IL-10, POMC, and PDYN was measured by using qRT-PCR. The data are presented as means ± SEM (*n* = 3 independent repeats with duplicates). The asterisk and number sign denote statistical significance (*p* < 0.001) compared to the control group and cinobufagin group, respectively, by unpaired and two-tailed Student *t* test (**a**–**f**) or one-way ANOVA followed by the post hoc Student–Newman–Keuls test (**g**, **h**)

In order to test its stimulatory effect on the expression of IL-10 and β-endorphin in cultured primary cells, cinobufagin (100 μM) was incubated for 2 h with microglia, neurons and astrocytes, originated from the spinal cords of neonatal rats. The gene expression of IL-10, POMC and prodynorphin from each set of cells was measured using qRT-PCR. As shown in Fig. 3a and b, treatment with cinobufagin for 2 h significantly stimulated the mRNA expression of IL-10 and POMC in cultured primary microglia (*p* < 0.001, by unpaired and two-tailed Student *t* test), but not in neurons or astrocytes. Further analysis showed that cinobufagin concentration-dependently increased the POMC expression in microglia with an EC_50_ value of 12.4 μM (Fig. 3c). In contrast, treatment with cinobufagin at 100 μM did not significantly alter the gene expression of PDYN in either cultured microglia, neurons, or astrocytes (Fig. 3d).

Moreover, the baseline IL-10 and β-endorphin levels in cultured microglia cells were 6.8 ± 0.9 and 49.2 ± 18.2 pg/mL. Exposure of cinobufagin (100 μM) significantly increased the protein expression of IL-10 by 13.6-fold and β-endorphin by 6.6-fold, respectively (*p* < 0.01 by unpaired and two-tailed Student *t* test; Fig. 3e, f).

To further confirm the stimulatory effect of cinobufagin on the microglial expression of IL-10 and POMC, the microglial inhibitor minocycline [55, 65] was used. Cultured microglial cells were treated with minocycline (60 μM) 1 h followed by cinobufagin (100 μM) and the gene expression of IL-10 and POMC was measured 2 h after treatment using qRT-PCR. Treatment with minocycline did not significantly alter the baseline expression of IL-10 or POMC, but its pretreatment significantly blocked cinobufagin-induced overexpression of IL-10 and POMC (*p* < 0.001, by one-way ANOVA followed by the post hoc Student–Newman–Keuls test; Fig. 3g, h).

**Fig. 4:**
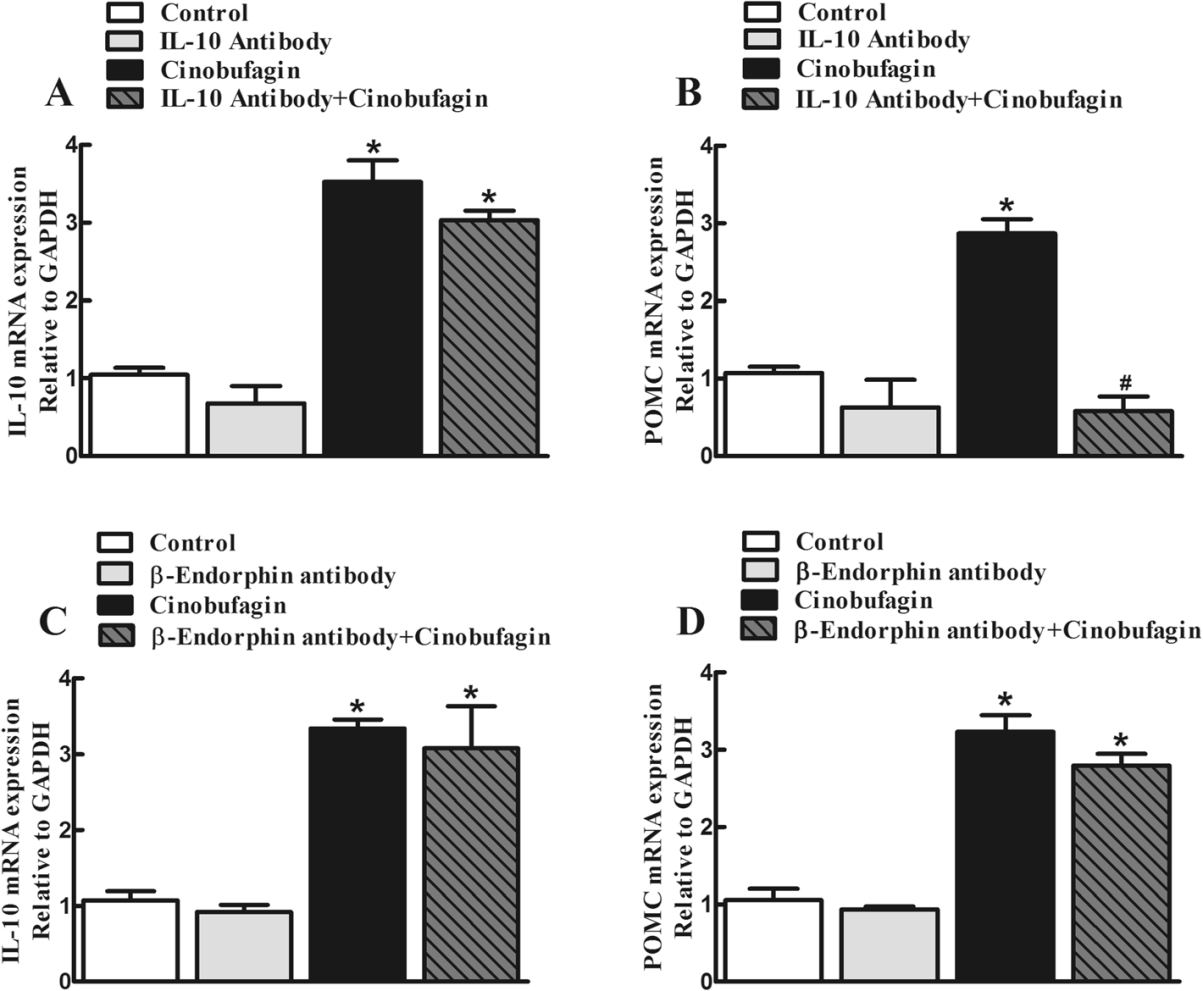
Effects of the IL-10 antibody (**a**, **b**) and β-endorphin antiserum (**c**, **d**) on cinobufagin-stimulated gene expression of IL-10 and β-endorphin precursor proopiomelanocortin (POMC) in primary cultures of spinal microglia. Microglia cells, originated from the spinal cords of 1-day-old neonatal rats, were first treated with the IL-10 antibody or β-endorphin antiserum for 2 h followed by cinobufagin, and were collected 2 h after the cinobufagin treatment. The mRNA expression of IL-10 and POMC was measured using qRT-PCR. The data are presented as means ± SEM (*n* = 3 independent repeats with duplicates). The asterisk denotes statistical significance (*p* < 0.001) compared to the control group, by unpaired and two-tailed Student *t* test

In order to reveal whether IL-10 induced β-endorphin expression or vice versa following cinobufagin treatment, the effects of the IL-10 and β-endorphin neutralizing antibodies were assessed on the gene expression of IL-10 and POMC. Cultured microglial cells were treated with the IL-10 antibody (4 μg/mL) or β-endorphin antiserum (1:300 dilution) 2 h followed by cinobufagin (100 μM) and the gene expression of IL-10 and POMC was measured 2 h later. As shown in Fig. 4a and b, treatment with cinobufagin significantly stimulated the gene expression of IL-10 and POMC, compared to the control group. Pretreatment with the IL-10 antibody for 2 h did not significantly alter baseline expression of IL-10 or POMC, but blocked cinobufagin-stimulated overexpression of POMC but not IL-10 (*p* < 0.0001, by one-way ANOVA followed by the post hoc Student–Newman–Keuls test). In contrast, treatment with the β-endorphin antiserum did not alter either the baseline or cinobufagin-stimulated gene expression of IL-10 or POMC (Fig. 4c, d).

### Cinobufagin exhibited mechanical antiallodynia through spinal microglial expression of IL-10 and β-endorphin

**Fig. 5:**
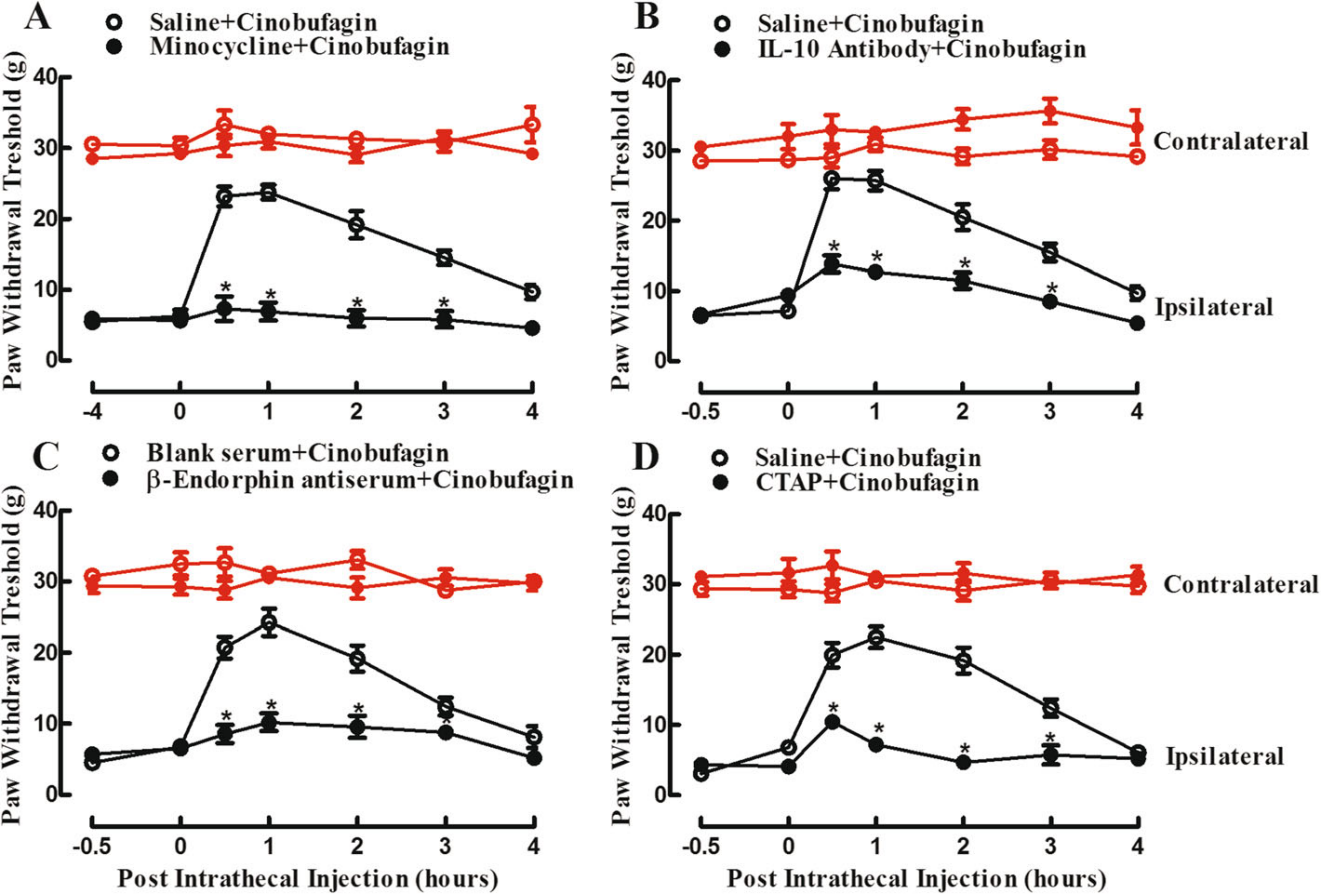
Blockade effects of intrathecal injection of the microglial inhibitor minocycline (**a**), IL-10 antibody (**b**), β-endorphin antiserum (**c**), and selective μ-opioid receptor CTAP (**d**) on spinal cinobufagin-induced mechanical antiallodynia in the rat model of bone cancer pain. Female bone cancer pain rats, approximately 3 weeks after cancer cell inoculation, received two intrathecal injections, and mechanical thresholds in both the contralateral and ipsilateral hindpaws were measured by using electric von Frey filaments. Minocycline was intrathecally injected 4 h prior to cinobufagin treatment, whereas the IL-10 antibody, β-endorphin antiserum, and CTAP were intrathecally given 30 min before cinobufagin injection. Withdrawal thresholds were measured in both the contralateral and ipsilateral hindpaws. The data are presented as means ± SEM (*n* = 6 per group). The asterisk denotes statistical significance (*p* < 0.05) compared to the vehicle control group, by repeated-measured two-way ANOVA followed by the post hoc Student–Newman–Keuls test

To assess whether cinobufagin exhibited antinociception through the spinal microglial IL-10/β-endorphin axis, the microglial inhibitor minocycline was first employed. The female bone cancer pain rats were divided into two groups and received intrathecal injection of normal saline (10 μL) or minocycline (100 μg) followed by cinobufagin (30 μg) 4 h later. Intrathecal injection of cinobufagin produced time-dependent mechanical antiallodynia in the ipsilateral hindpaws. Pretreatment with intrathecal injection of minocycline did not significantly affect baseline withdrawal thresholds in the ipsilateral hindpaws, but completely blocked cinobufagin-induced mechanical antiallodynia (*p* < 0.0001, by repeated-measures two-way ANOVA followed by the post hoc Student–Newman–Keuls test; Fig. 5a).

In addition, two groups of female bone cancer pain rats received intrathecal injection of 10 μL of normal saline or 2 μg of the IL-10 antibody followed by 30 μg of cinobufagin 30 min later. Intrathecal injection of cinobufagin in the ipsilateral hindpaws produced a time-dependent mechanical antiallodynia, which was significantly attenuated by the pretreatment with intrathecal injection of the IL-10 antibody (*p* < 0.0001, by repeated-measures two-way ANOVA followed by the post hoc Student–Newman–Keuls test) although it did not significantly alter the baseline withdrawal thresholds (Fig. 5b).

Furthermore, two groups of female bone cancer pain rats received intrathecal injection of normal saline (10 μL) or the β-endorphin antiserum (1:10 dilution, 10 μL) followed by cinobufagin (30 μg) 30 min later. The pretreatment with intrathecal injection of the β-endorphin antiserum did not have a significant effect on the baseline withdrawal thresholds, but effectively attenuated cinobufagin-induced mechanical antiallodynia (*p* < 0.0001, by repeated-measures two-way ANOVA followed by the post hoc Student–Newman–Keuls test; Fig. 5c).

As β-endorphin is an endogenous ligand of μ-opioid receptors [32, 44], its involvement in cinobufagin-induced mechanical antiallodynia was investigated. Two groups of female bone cancer pain rats received intrathecal injection of normal saline (10 μL) or the selective μ-opioid receptor antagonist CTAP (10 μg) followed by cinobufagin (30 μg) 30 min later. As shown in Fig. 5d, the pretreatment with intrathecal injection of CTAP effectively blocked the antinociceptive effect of cinobufagin in the ipsilateral hindpaws (*p* < 0.0001, by repeated-measures two-way ANOVA followed by the post hoc Student–Newman–Keuls test), although it did not significantly affect the baseline withdrawal thresholds.

### Activation of the α7-nAChR mediated cinobufagin-induced spinal microglial expression of IL-10/β-endorphin and mechanical antiallodynia

**Fig. 6:**
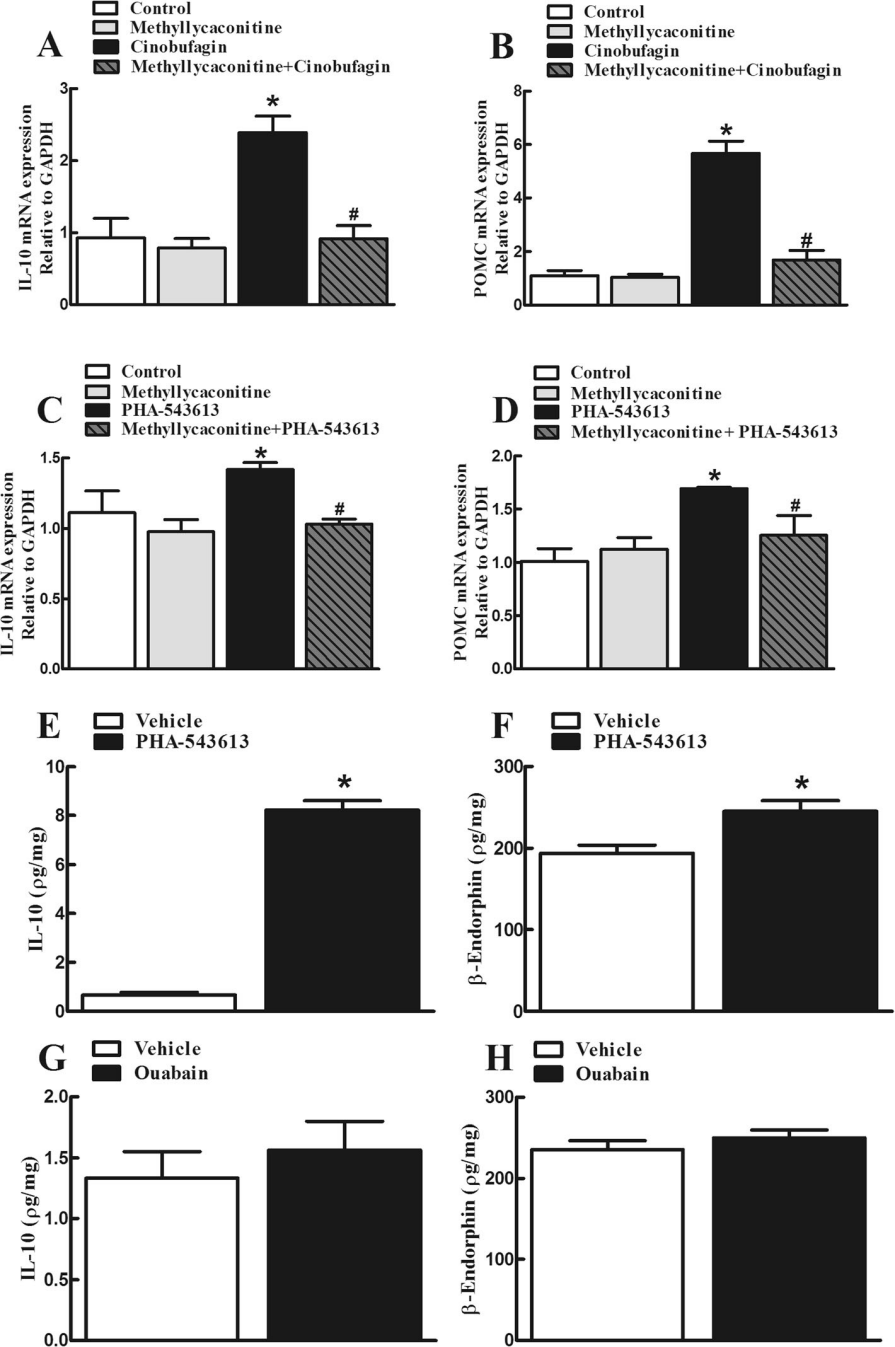
Blockade effects of the specific α7-nicotinic acetylcholine receptor (α7-nAChR) antagonist methyllycaconitine on cinobufagin (**a**, **b**)- and the specific α7-nAChR agonist PHA-543613 (**c**, **d**)-stimulated gene expression of IL-10 and the β-endorphin precursor proopiomelanocortin (POMC) in primary cultures of spinal microglia. **e**–**h**. Effects of PHA-543613 and ouabain on the protein expression of IL-10 and β-endorphin. Microglial cells, originated from the spinal cords of 1-day-old neonatal rats, were incubated with cinobufagin (100 μM), PHA-543613 (100 μM), or ouabain (10 μM). The mRNA and protein expression of IL-10 and POMC was measured 2 h later using qRT-PCR and the commercial kits, respectively. For the methyllycaconitine blockade study, methyllycaconitine was incubated 0.5 h prior to cinobufagin or PHA-543613 treatment. The data are presented as means ± SEM (*n* = 3 independent repeats with duplicates). The asterisk and number sign denote statistical significance (*p* < 0.001) compared to the control and cinobufagin group, respectively, by one-way ANOVA followed by the post hoc Student–Newman–Keuls test

To test whether activation of the α7-nAChR was responsible for cinobufagin-stimulated microglial expression of IL-10 and β-endorphin, the specific α7-nAChR antagonist methyllycaconitine [6] was employed. Cultured primary microglial cells, originated from the spinal cords of neonatal rats, were treated with methyllycaconitine (10 nM) [76] 30 min later followed by cinobufagin (100 μM), and the gene expression of IL-10 and POMC was measured 2 h after the treatment using qRT-PCR. Methyllycaconitine treatment did not significantly alter the baseline IL-10 or POMC expression in microglia, but its pretreatment entirely blocked cinobufagin-induced overexpression of IL-10 and POMC (*p* < 0.001, by one-way ANOVA followed by the post hoc Student–Newman–Keuls test; Fig. 6a, b).

To further confirm the stimulatory effects of α7-nAChR activation on the IL-10 and β-endorphin expression, the specific α7-nAChR agonist PHA-543613 [89] was also used in the study. Cultured spinal primary microglial cells were treated with methyllycaconitine (10 nM) 30 min later followed by PHA-543613 (100 μM), and the gene expression of IL-10 and POMC was measured 2 h later using qRT-PCR. As shown in Fig. 6c and d, treatment with PHA-543613 increased the gene expression of IL-10 and POMC, which was blocked by the pretreatment with methyllycaconitine (*p* < 0.05, by one-way ANOVA followed by the post hoc Student–Newman–Keuls test). In addition, treatment with PHA-543613 (100 μM) for 2 h significantly increased the protein expression of IL-10 and β-endorphin in primary cultures of microglia (*p* < 0.0001, by unpaired and two-tailed Student *t* test; Fig. 6e and f). To investigate whether the stimulatory effect of cinobufagin on the IL-10 and β-endorphin expression was possibly via the inhibition of Na^+^-K^+^ ATPase, the Na^+^-K^+^ ATPase inhibitor ouabain [37] was tested. As shown in Fig. 6g and h, treatment with ouabain (10 μM) failed to affect the protein expression of IL-10 or β-endorphin in primary cultures of microglia.

**Fig. 7:**
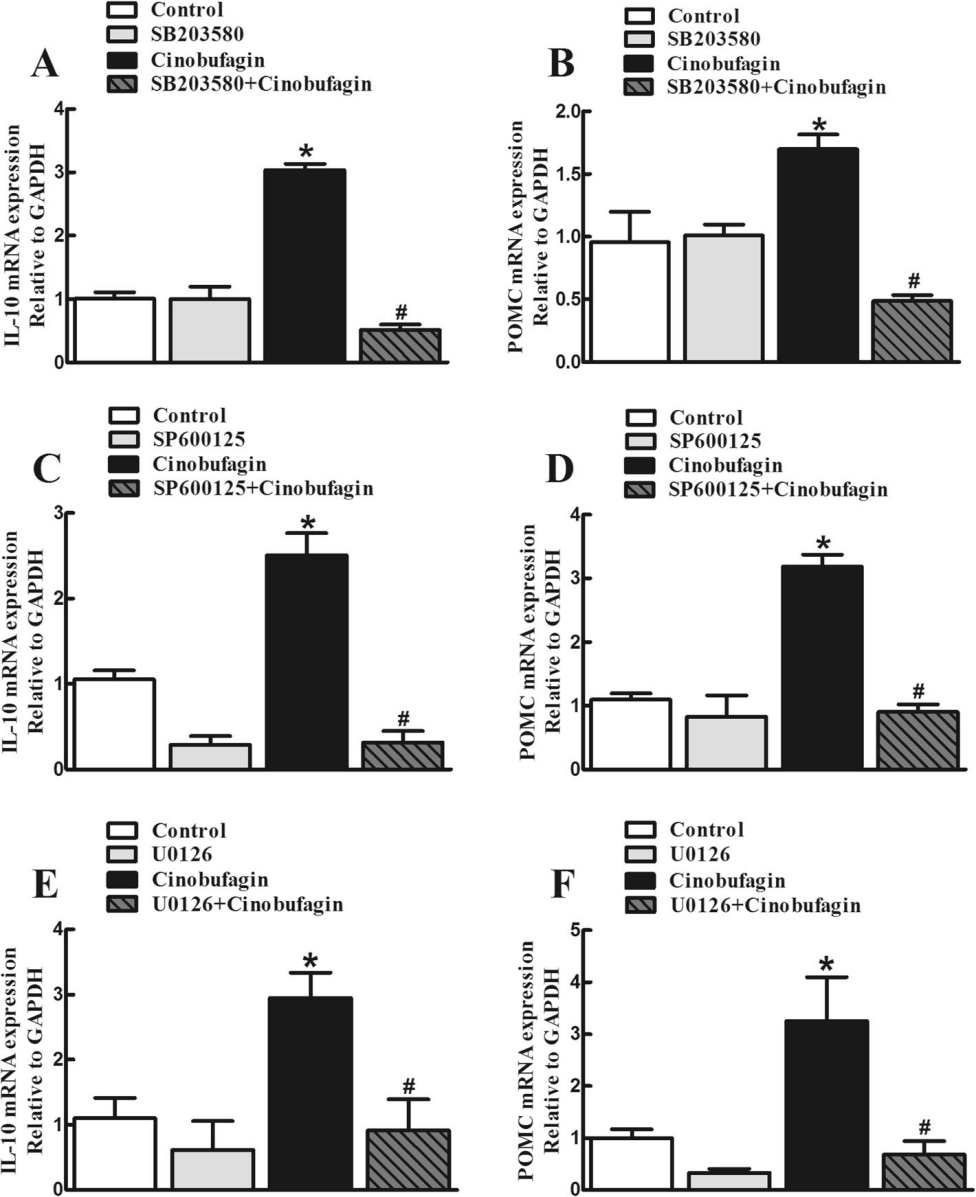
Blockade effects of the specific p38 mitogen-activated protein kinase (MAPK) activation inhibitor SB203580 (**a**, **b**), JNK MAPK activation inhibitor SP600125 (**c**, **d**), and ERK1/2 MAPK activation inhibitor UO126 (**e**, **f**) on cinobufagin-stimulated gene overexpression of IL-10 and the β-endorphin precursor proopiomelanocortin (POMC) in primary cultures of spinal microglia. Cultured primary microglial cells, originated from the spinal cords of 1-day-old neonatal rats, incubated with each MAPK inhibitor for 1 hour followed by cinobufagin (100 μM) for 2 h. The mRNA expression of IL-10 and POMC was measured using qRT-PCR. The data are presented as means ± SEM (*n* = 3 independent repeats with duplicates). The asterisk and number sign denote statistical significance (*p* < 0.001) compared to the control group and cinobufagin group, respectively, by one-way ANOVA followed by the post hoc Student–Newman–Keuls test

Activation of mitogen-activated protein kinases (MAPKs), including p38, extracellular signal-related kinases 1/2 (ERK1/2) and c-Jun N-terminal kinases (JNK) [75], was involved in α7-nAChR-induced inhibition of neuroinflammation [23, 49, 85]. To illustrate which subtype(s) of MAPKs were involved in cinobufagin-induced IL-10/β-endorphin expression, the specific p38 inhibitor SB203580, JNK inhibitor SP600125, and ERK1/2 inhibitor UO126 were used. Cultured microglial cells were treated with SB203580 (30 μM), SP600125 (50 μM), or UO126 (50 μM) 30 min followed by cinobufagin (100 μM), and the gene expression of IL-10 and POMC was measured 2 h after cinobufagin treatment using qRT-PCR. As shown, treatment with SB203580 (Fig. 7a, b), SP600125 (Fig. 7c, d), or UO126 (Fig. 7e, f) did not significantly alter baseline gene expression of IL-10 or POMC. However, each treatment significantly inhibited cinobufagin-induced overexpression of IL-10 and POMC (*p* < 0.0001, by one-way ANOVA followed by the post hoc Student–Newman–Keuls test).

**Fig. 8:**
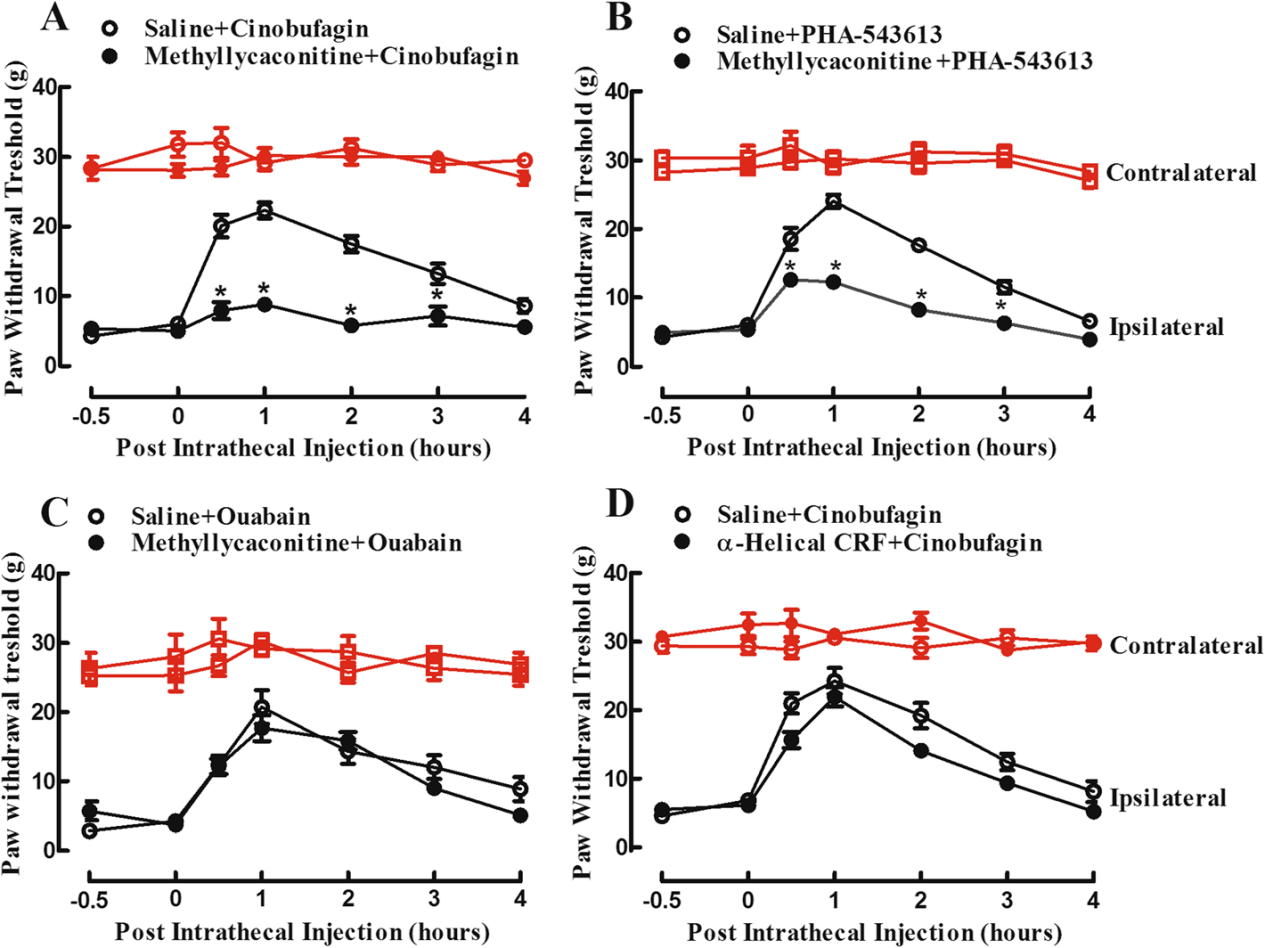
Effects of the specific α7-nicotinic acetylcholine receptor (α7-nAChR) antagonist methyllycaconitine (**a**–**c**) and CRF receptor antagonist α-helical CRF (9-41) (**d**) on cinobufagin-, PHA-543613-, or ouabain-induced mechanical antiallodynia in the rat model of bone cancer pain. Female bone cancer pain rats, approximately 3 weeks after cancer cell inoculation, received intrathecal injection of the vehicle (10 μL), methyllycaconitine (10 μg) or α-helical CRF (9-41) (20 μg) 0.5 h later followed by cinobufagin (30 μg), PHA-543613 (12 μg), or ouabain (2.5 μg), and mechanical thresholds in both the contralateral and ipsilateral hindpaws were measured by using electric von Frey filaments. The data are presented as means ± SEM (*n* = 6 per group). The asterisk denotes statistical significance (*p* < 0.05) compared to the vehicle control group, by repeated-measured two-way ANOVA followed by the post hoc Student–Newman–Keuls test

Futhermore, the inhibitory effect of methyllycaconitine on cinobufagin-induced mechanical antiallodynia was assessed. Two groups of female bone cancer pain rats received intrathecal injection of normal saline (10 μL) or methyllycaconitine (10 μg) followed by cinobufagin (30 μg) 30 min later, and withdrawal thresholds were measured in both the contralateral and ipsilateral hindpaws prior to or post the last injection. As shown in Fig. 8a, intrathecal injection of methyllycaconitine did not significantly alter baseline withdrawal thresholds in either contralateral or ipsilateral hindpaws, whereas cinobufagin produced marked mechanical antiallodynia in the ipsilateral hindpaws. Pretreatment with methyllycaconitine blocked cinobufagin-induced mechanical antiallodynia (*p* < 0.0001, by repeated-measures two-way ANOVA followed by the post hoc Student–Newman–Keuls test).

For comparison, the mechanical antiallodynic effects of PHA-543613 and ouabain were also assessed. Four groups of female bone cancer pain rats received intrathecal injection of normal saline (10 μl) or methyllycaconitine (10 μg) followed by an intrathecal injection of PHA-543613 (12 μg) or ouabain (2.5 μg) 30 min later [90, 92]. Intrathecal injection of PHA-543613 significantly inhibited mechanical allodynia in the ipsilateral hindpaws, without affecting mechanical thresholds in the contralateral hindpaws. Pretreatment with intrathecal injection of methyllycaconitine blocked PHA-543613-induced mechanical antiallodynia (*p* < 0.0001, by repeated-measures two-way ANOVA followed by the post hoc Student–Newman–Keuls test; Fig. 8b). In contrast, intrathecal injection of ouabain exerted a time-dependent mechanical antiallodynia, which was not significantly altered by the pretreatment with intrathecal injection of methyllycaconitine (Fig. 8c). No obvious behavioral changes of ouabain were observed during the study. However, intrathecal injection of more than 5 μg of ouabain produced abnormal behavior changes such as shortness of breath and tonic convulsion.

The CRF receptor was associated with microglial expression of β-endorphin [8, 33, 40] and *Bufonis venenum* stimulated CRF expression [9, 10]. Thus, the possible involvement of the CRF receptor in cinobufagin-induced mechanical antiallodynia was finally tested. Two groups of female bone cancer pain rats received intrathecal injection of normal saline (10 μL) and the specific CRF receptor antagonist α-helical CRF (9-41) (20 μg) [32, 59] respectively followed by cinobufagin (30 μg) 30 min later, and withdrawal thresholds were measured in both the contralateral and ipsilateral hindpaws prior to or post the last injection. In contrast to methyllycaconitine, pretreatment with intrathecal injection of α-helical CRF (9-41) failed to block the antiallodynic effect of cinobufagin in the ipsilateral hindpaws (Fig. 8d).

## Discussion

Cancer pain particularly bone cancer pain remains to be one of the most challenging symptoms to control [36, 51]. Current major drug treatment for advanced cancer pain is strong opioids, such as morphine, methadone, oxycodone, hydromorphone, and fentanyl [26]. However, the treatment success rate is still low especially with tremendous adverse effects, including oversedation, respiration inhibition, constipation, analgesic tolerance and hyperalgesia, and physical dependence and addiction. It is thus critical to discover and develop novel non-addictive, highly effective and low-toxicity interventions for the treatment of cancer pain. *Bufonis venenum* has been prescribed in China for the treatment of cancers [2, 12]. Since it was serendipitously found to effectively relieve pain in bone metastasis patients during anticancer treatment [50], *Bufonis venenum* has been frequently used to treat a variety of cancer pains as it also has anticancer effects [22, 50]. The clinical analgesic efficacy was supported by the findings that multiple daily intraperitoneal injections of *Bufonis venenum* produced mechanical antiallodynia and thermal antihyperalgesia in the mouse model of paw cancer pain [9, 10]. Our current study demonstrated that intrathecal injection of cinobufagin, probably the most abundant bufadienolide in *Bufonis venenum*, inhibited mechanical allodynia in the ipsilateral hindpaws of female bone cancer rats in a dose-dependent manner, with an *E*_max_ of 90% MPE and ED_50_ of 6.4 μg. However, intrathecal cinobufagin at the doses tested up to 100 μg did not affect normal mechanical thresholds in the contralateral hindpaws. Accumulated evidence indicates that gender is an important factor in pain modulation and the sex hormones difference influences the response to opioids for pain treatment [19, 57]. Thus, the mechanical antiallodynic effects of cinobufagin were also assessed in male cancer pain rats. Our result demonstrated that intrathecal injection of cinobufagin similarly inhibited mechanical allodynia in the ipsilateral hindpaws of male bone cancer rats. Therefore, all the results confirm that *Bufonis venenum* and its derived cinobufagin are effectively analgesic in bone cancer pain and provide a new molecular scaffold of cinobufagin for chemical modification and development as a painkiller for cancer pain.

More strikingly, the present study reveals that cinobufagin induces mechanical antiallodynia through the spinal microglial IL-10/β-endorphin pathway. This notion is supported by the following findings: (1) Treatment with cinobufagin in cultured primary spinal microglial cells, but not neurons or astrocytes, specifically stimulated the gene and protein expression of IL-10 and POMC but not PDYN, which was inhibited by the pretreatment with the microglial inhibitor minocycline. In addition, treatment with cinobufagin also stimulated other M2 biomarkers Arg1, CD206, and IL-4 in cultured microglia (unpublished data). Moreover, pretreatment with the IL-10 antibody prevented cinobufagin-induced stimulatory effect on the gene expression of POMC but not IL-10; whereas pretreatment with the β-endorphin antiserum failed to affect its stimulatory effect on the mRNA expression of either IL-10 or POMC. The results indicated that cinobufagin-stimulated microglial expression of IL-10 and subsequent POMC and not vice versa. The conclusion is supported by the recent findings that IL-10 treatment directly stimulated microglial expression of β-endorphin, whereas β-endorphin treatment did not have a significant stimulatory effect on IL-10 expression [71, 77–79]. Furthermore, pretreatment with the IL-10 antibody prevented the GLP-1 receptor agonist exenatide- and GPR40 agonist GW90851-induced expression of β-endorphin but not IL-10, whereas the β-endorphin antiserum did not have any effects on their expression of β-endorphin or IL-10 [52, 79]. (2) Intrathecal injection of cinobufagin significantly stimulated the gene and protein expression of IL-10 and β-endorphin but not dynorphin A in the ipsilateral spinal cords. The results were in agreement with the previous findings that multiple daily intraperitoneal injections of *Bufonis venenum* increased the β-endorphin level in plasma and β-endorphin immunostaining in the tumor xenograft tissues of paw cancer pain mice [9, 10]. (3) Causally, the spinal cinobufagin-induced mechanical antiallodynia was nearly entirely attenuated by the pretreatment with intrathecal injection of the microglial inhibitor minocycline, IL-10 neutralizing antibody, β-endorphin antiserum, and specific μ-opioid receptor antagonist CTAP. Our previous study also demonstrated that intrathecal IL-10 in neuropathic rats produced mechanical antiallodynia and thermal antihyperalgesia, which were prevented by the pretreatment with the β-endorphin antiserum and CTAP [55, 56, 77, 78].

Expressed in astrocytes and microglial cells in addition to neurons, α7-nAChRs are closely associated with anti-neuroinflammation through the cholinergic nervous system and may be a promising target molecule for the treatment Alzheimer’s disease, schizophrenia, Parkinson disease, and chronic pain [1, 14, 27, 38, 62, 66, 83]. It was recently reported that gavage administration of cinobufagin produced antinociception in the mouse models of thermal and chemical pain, which was prevented by the intraperitoneal injection of the specific α7-nAChR antagonist methyllycaconitine and intrathecal injection of the gene silencer siRNA/α7-nAChR [82]. The present study confirmed that intrathecal injection of methyllycaconitine in the rat model of bone cancer pain blocked spinal cinobufagin-induced mechanical antiallodynia and pretreatment with methyllycaconitine in cultured primary microglial cells attenuated cinobufagin-stimulated gene expression of IL-10 and POMC. Furthermore, treatment with the specific α7-nAChR agonist PHA-543613 in cultured primary microglia stimulated gene and protein expression of IL-10 and β-endorphin, and intrathecal PHA-543613 in bone cancer pain rats produced mechanical antiallodynia, both of which were blocked by the pretreatment with methyllycaconitine. These results suggest that cinobufagin and PHA-543613 activate α7-nAChRs to stimulate spinal microglial expression of IL-10 and β-endorphin and produce mechanical antiallodynia in bone cancer pain. However, this study is limited by lacking elucidated interactions of cinobufagin with the α7-nAChR at the molecular level. Further studies are needed to determine the efficiency and mode of cinobufagin in the in vitro experiments (such as radioligand analysis, electrophysiology or calcium imaging FLIPR assay) with α7-nAChRs expressed in the Xenopus oocytes or appropriate cell lines. On the other hand, CRF receptors are also associated with microglial expression of β-endorphin [8, 33, 40] and *Bufonis venenum* was reported to stimulate CRF expression [9, 10]. However, our study showed that the CRF receptor antagonist α-helical CRF (9-41) was ineffective in blockade of cinobufagin-induced antiallodynia, implying that the spinal CRF system may not mediate cinobufagin-induced β-endorphin expression via IL-10 and subsequent mechanical antiallodynia.

Cinobufagin and its analogs inhibited Na^+^-K^+^ ATPase [11, 17, 18], which might also be the mechanisms accounting for their antinociception as inhibition of Na^+^-K^+^ ATPase leads to antinociception [15, 68]. Indeed, intrathecal treatment with ouabain in bone cancer pain produced marked mechanical antiallodynia which was not reversed by the α7-nAChR antagonist. However, microglia do not express functional Na^+^-K^+^ ATPase, and H^+^/K^+^-ATPase instead appears to vicariate its role in microglia [5, 61]. It was true that treatment with ouabain did not stimulate the expression of IL-10 and β-endorphin in primary microglial cells in our study. Thus, the stimulatory effect of cinobufagin on IL-10 and β-endorphin expression may not be due to its inhibition of Na^+^-K^+^ ATPase [4, 16]. Taken together, all these results suggest that cinobufagin produces mechanical antiallodynia by activating α7-nAChRs, rather than inhibition of Na^+^-K^+^ ATPase, and that microglial activation of α7-nAChRs triggers the IL-10/β-endorphin antinociceptive pathway in painful hypersensitivity states. On the other hand, *Bufanis venenum* and bufadienolide have been reported to exhibit various side-effects or toxicities in the central nervous and cardiovascular systems, such as shortness of breath, seizure, tachecardia, cardiac arrhythmia, and even coma [70, 81, 82];). Truly, the behavioral toxicities such as shortness of breath, restless movements, and tonic convulsion were observed after the intrathecal injection of more than 250 μg of cinobufagin or more than 5 μg of ouabain. The side-effects of cinobufagin are probably due to inhibition of Na^+^-K^+^ ATPase expressed in neurons and cardiocytes ([11, 17, 18, 20, 70]. It is thus suggested that *Bufanis venenum*- and its effective ingredients-induced side-effects or toxicities are separated from their antinociception.

**Fig. 9:**
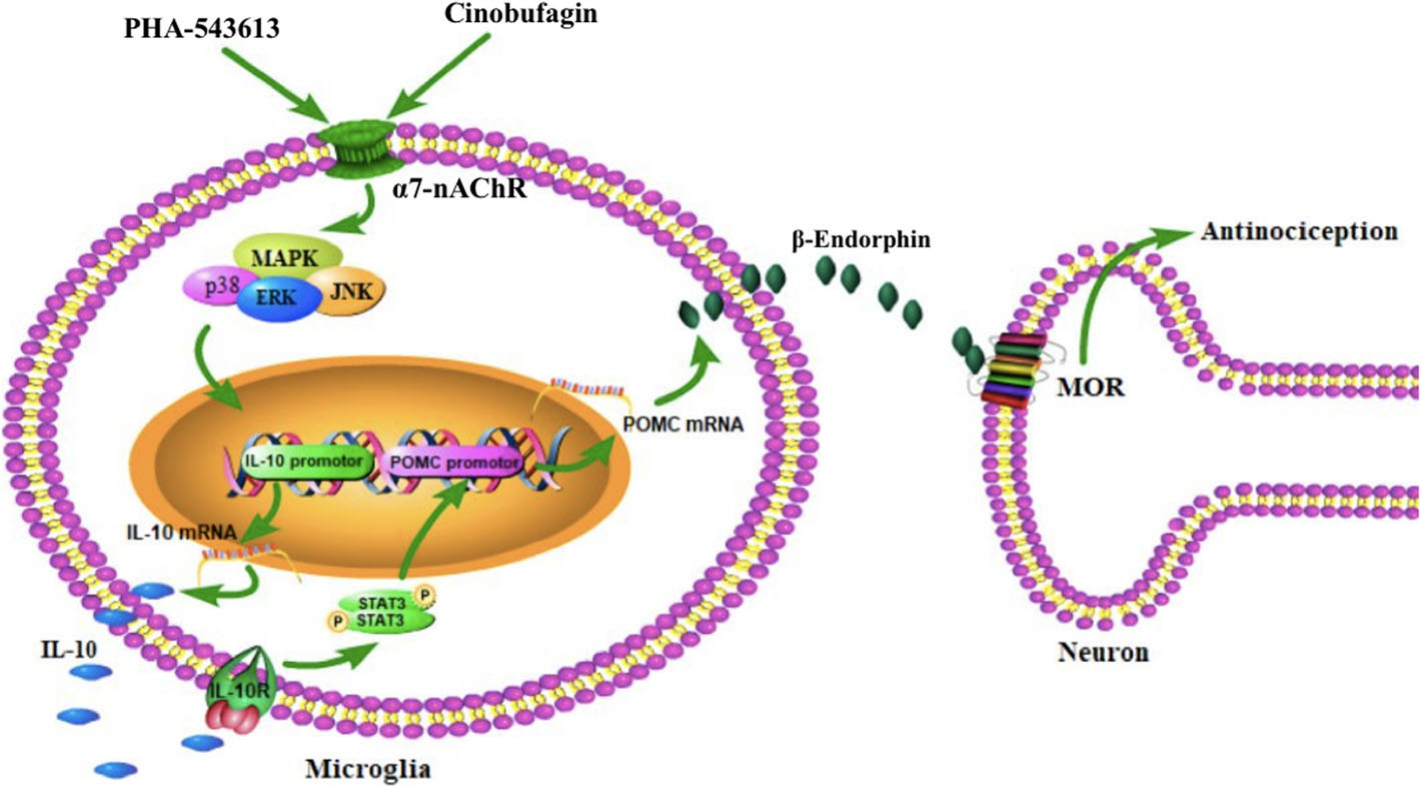
Illustration of the proposed mechanisms underlying cinobufagin-mediated antinociception through stimulation of spinal microglial IL-10/β-endorphin pathway following α7-nicotinic acetylcholine receptor (α7-nAChR) activation. Cinobufagin and PHA-543613 stimulate mitogen-activated protein kinase (MAPK) activation following agonism of the α7-nAChR and excrete IL-10, which subsequently activates the IL-10 receptor and expresses β-endorphin through an autocrine mechanism. The released β-endorphin passes the microglial neuronal synapse and activates μ-opioid receptor (MOR) in neurons to produce antinociception

The notion that agonism of microglial α7-nAChR triggers IL-10/β-endorphin pathway is further supported by the facts that the specific inhibitors of MAPK activation attenuated cinobufagin-stimulated IL-10/β-endorphin expression in microglial cells. MAPKs are a family of evolutionally conserved molecules including p38, JNK, and ERK1/2 [75] and have been suggested to be involved in the antiinflammatory properties of α7-nAChRs possibly through modulation of inflammatory cytokine release [23, 49, 60, 69, 85]. Our study demonstrated that the specific JNK activation inhibitor SP600125, ERK1/2 activation inhibitor UO126 and p38 activation inhibitor SB203580 significantly blocked the gene expression of IL-10 and POMC in cultured microglia. The results agree with previous findings in which each subtype of MAPKs was involved in the production of IL-10 in human alveolar macrophages and monocytes [7, 28, 29, 39, 58, 63, 64, 93]. These results suggest that each ERK1/2, JNK, and p38 MAPK activation is associated with cinobufagin-induced expression of IL-10 after activation of α7-nAChRs. Furthermore, activation of α7-nAChR has been correlated with JAK-STAT pathway [13, 94]. Our recent findings indicated that exogenous and endogenous IL-10 stimulated β-endorphin expression via the IL-10 receptor/JAK1/STAT3 signal pathway by the autocrine mechanism [77–79]. Thus, we postulate that cinobufagin produces mechanical antiallodynia through microglial expression of IL-10 following α7-nAChR activation and MAPK signaling, followed by expression of β-endorphin via the IL-10 receptor/JAK1/STAT3 signaling. The pathway for cinobufagin to produce mechanical antiallodynia in bone cancer pain is schematically illustrated in Fig. 9.

It has been extensively shown that activation of microglia is associated with the development of neurodestruction and nociception [3, 84, 86]. However, accumulated evidence reveals that microglia also plays an important role in neuroprotection and antinociception [21, 30, 34, 45, 77, 78]. Expressed in astrocytes and microglia, IL-10 is a known antiinflammatory and immunosuppressive cytokine and exhibits marked antinociception in neuropathic pain and inflammatory pain [42, 54, 67, 77, 78]. We have recently discovered that IL-10 produced antinociception through spinal microglial expression of β-endorphin [77, 78], by which activation of the GLP-1 receptor and GPR40 produced mechanical antiallodynia and thermal antihyperalgesia in a variety of rodent models of painful hypersensitivity [52, 79]. Our current cinobufagin findings further highlight the broad significance of the recently uncovered spinal microglial IL-10/β-endorphin pathway in the regulation of antinociception in chronic pain.

## Conclusion

IL-10, expressed in astrocytes and microglia, is a known antiinflammatory and immunosuppressive cytokine and exhibits marked antinociception in neuropathic pain and inflammatory pain. We have recently discovered that IL-10 produced antinociception through spinal microglial expression of β-endorphin, by which activation of the GLP-1 receptor and GPR40 produced mechanical antiallodynia and thermal antihyperalgesia in a variety of rodent models of painful hypersensitivity. In this study, we postulate that cinobufagin produces mechanical antiallodynia through microglial expression of IL-10 after α7-nAChR activation and MAPK signaling, followed by expression of β-endorphin. Our cinobufagin findings further highlight the broad significance of the recently uncovered spinal microglial IL-10/β-endorphin pathway in the regulation of antinociception in chronic pain.

## Data Availability

All data supporting the conclusion of the article are included in this article.

## Abbreviations

IL: Interleukin
TNF: Tumor necrosis factor
α7-nAChR: α7-nicotinic acetylcholine receptor
CRF: Corticotropin-releasing factor
GLP-1: Glucagon-like peptide-1
POMC: Proopiomelanocortin
PDYN: Prodynorphin
MAPK: Mitogen-activated protein kinase
ERK1/2: Extracellular signal-related kinase 1/2
JNK: c-Jun N-terminal kinase
MPE: Maximal possible effect
